# A Preliminary Investigation of the Efficacy of Far-Infrared-Emitting Garments in Enhancing Objective and Subjective Recovery Following Resistance Exercise

**DOI:** 10.3390/jfmk10030280

**Published:** 2025-07-18

**Authors:** Jonathon R. Lever, Cara Ocobock, Valerie Smith-Hale, Casey J. Metoyer, Alan Huebner, John P. Wagle, Jonathan D. Hauenstein

**Affiliations:** 1 Sports Performance, University of Notre Dame, Notre Dame, IN 46556, USA; vsmith4@nd.edu (V.S.-H.); casey.metoyer@gmail.com (C.J.M.); jwagle@nd.edu (J.P.W.); 2 Department of Anthropology, University of Notre Dame, Notre Dame, IN 46556, USA; cocobock@nd.edu; 3 Department of Applied and Computational Mathematics and Statistics, University of Notre Dame, Notre Dame, IN 46556, USA; alan.huebner.10@nd.edu

**Keywords:** countermovement jump, neuromuscular performance, recreational athletes

## Abstract

**Objective:** This study aimed to investigate the efficacy of far-infrared (FIR) garments in enhancing recovery following resistance exercise in recreationally active individuals. **Methods:** Ten recreationally active adults (six females, four males; aged 20.7 ± 3.2 years) completed a resistance exercise protocol and were randomly selected to wear either FIR (n = 5) or placebo (n = 5) tights post-exercise. The FIR garments incorporated Celliant-based fibers emitting wavelengths in the 2.5–20 µm range. The participants’ recovery was assessed using countermovement jump (CMJ) metrics, including their jump height, takeoff velocity, and modified reactive strength index (mRSI), along with their fatigue biomarkers and subjective recovery perceptions. The CMJ performance was tested immediately post-exercise and at 24 and 48 h. **Results:** The FIR garments led to significant improvements in neuromuscular recovery, with greater increases in the jump height, takeoff velocity, and mRSI observed at 48 h post-exercise (*p* < 0.05). Notably, the mRSI showed earlier improvements at 24 h. The fatigue biomarkers did not differ between the groups (*p* > 0.05), suggesting localized rather than systemic recovery effects. The participants in the FIR group reported faster subjective recovery, with a readiness to resume activity perceived within 48 h, compared to slower recovery in the placebo group. **Conclusions:** FIR garments may enhance neuromuscular recovery and subjective recovery perceptions following resistance exercise, likely by improving the peripheral blood flow, metabolic clearance, and tissue oxygenation. These findings suggest that FIR garments may be effective in enhancing both neuromuscular and perceived recovery following resistance exercise, supporting their potential use as a post-exercise recovery tool.

## 1. Introduction

The pursuit of noninvasive and convenient post-exercise recovery methods is of growing interest, particularly among active individuals who balance physical activity with work, education, or other responsibilities. Traditional recovery strategies include the use of NSAIDs, cold or heat therapy, stretching, massage, and nutritional supplements, alongside more advanced modalities such as ultrasound, vibration, lasers, and infrared (IR) therapies [1,2,3]. While these methods can alleviate delayed-onset muscle soreness (DOMS), their effects are often modest in terms of their ability to restore muscle function [4]. Many of these approaches also require a significant amount of time or access to specialized facilities, posing challenges for those with competing demands. There is a clear need for accessible, time-efficient recovery options that do not rely on medications or extensive infrastructure.

Far-infrared (FIR) garments have emerged as a potential alternative, offering convenience alongside potential physiological benefits. Constructed from fabrics embedded with FIR-emitting polymers or ceramic nanoparticles, these garments reflect the body’s natural FIR waves (3–30 µm) back into its tissues, with these waves able to penetrate up to 4 cm and interact with water molecules. This interaction causes the vibrational excitation of water molecules within the tissues, increasing their kinetic energy and promoting localized warming [5,6]. This thermal effect is thought to enhance capillary dilation, increase the blood flow, and stimulate mitochondrial activity [7,8]. These changes may support several key processes involved in recovery from exercise, including the removal of metabolic byproducts (e.g., lactate), a reduction in inflammation, and improved oxygen and nutrient delivery to damaged tissues [7,8]. The garments used in this study emitted FIR wavelengths ranging from 2.5 to 20 µm, with a power output of 338.0 mW/m^2^ and an emissivity of 0.888 at 35 °C. The participants wore the garments for approximately 12 h per day over two days, allowing for sustained exposure during the key phases of post-exercise recovery [8]. This process is believed to enhance the blood flow, reduce inflammation, and stimulate cellular metabolism [5,6]. While FIR technology has long been employed in medical contexts to manage chronic pain, inflammation, and arthritis and promote wound healing [7,9], its potential for improving exercise recovery is relatively underexplored.

Initial studies on FIR lamps have demonstrated promising effects, accelerating strength recovery and reducing muscle soreness and creatine kinase levels following eccentric exercise [4]. For example, Chen et al. (2023) [4] measured strength recovery using the maximal voluntary isometric contraction at multiple time points up to 72 h post-exercise in trained males. Meanwhile, FIR saunas have been shown to improve explosive performance measures, such as countermovement jump height, after endurance exercise [10], although not all studies have found advantages over traditional saunas [11]. However, the quality and emission spectrum of FIR lamps and saunas can vary widely, with some using low-emissivity materials or an insufficient thermal output, which may reduce their physiological efficacy [6]. Similarly, research on FIR garments has reported mixed outcomes. Some studies suggest improvements in muscle oxygenation, blood flow, lactate clearance, and perceived recovery [12,13,14], while others have found limited benefits, with the effects falling short of statistical significance despite moderate-to-large effect sizes [8,15]. Prior studies typically used ceramic-infused garments (e.g., shirts, tights) embedded with bioceramic or Celliant-based fibers emitting FIR waves in the 4–20 µm range. The inconclusive results may stem from small sample sizes, varied exercise responses, and a lack of standardization in the garment composition.

Given the limited and conflicting evidence, particularly in active populations, the present study aimed to investigate the effects of FIR-emitting tights on recovery following resistance exercise. Specifically, we sought to determine whether these garments can enhance recovery as assessed by the countermovement jump (CMJ) performance, subjective soreness, and fatigue-related biomarkers. Therefore, the objective of this study was to evaluate the efficacy of far-infrared (FIR)-emitting garments in enhancing neuromuscular and subjective recovery following resistance exercise in recreationally active individuals.

## 2. Materials and Methods

This article follows the STROBE cross-sectional reporting guidelines [16].

### 2.1. Participants

The participants in this study included ten healthy adults (six females, four males) who were all active members of the University Powerlifting Club. This participant population was chosen due to their familiarity and prior experience with weightlifting movements. Individuals with musculoskeletal injuries, known cardiovascular or metabolic disorders, or recent illnesses were not eligible to participate. The following protocol was reviewed and approved by the University of Notre Dame (Protocol 23-08-8032, 19 March 2024).

### 2.2. Study Design

**Figure 1:**
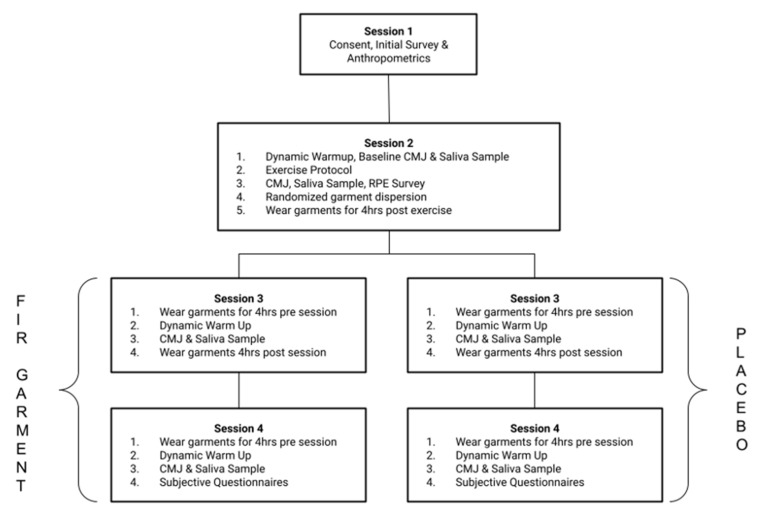
Diagrammatic representation of the method.

A double-blind randomization protocol was implemented to conduct this study, whereby the participants and the researchers collecting the data did not know which tights were FIR-emitting and which were the placebo. One investigator not involved in data collection or analysis coded the tights to ensure the researchers remained blinded. Data collection took place over 4 sessions (see Figure 1), each between 9 am and 12 pm. Session one consisted of the consent process, a survey, and anthropometry, while the remaining three sessions involved the resistance exercise protocol, saliva collection, and CMJs. Each participant took part in all the sessions, with 2 weeks between the sessions. Half of the participants were randomly assigned the FIR garments, with the remaining participants provided with the placebo. The FIR garment (KYMIRA, Reading, UK) consisted of 50% medical-grade KYnergy Polyester powered by Celliant, 25% Polyester, and 25% Spandex. It weighed 275 g/m^2^ and emitted waves with a power of 338.0 mW/m^2^, an emissivity of 0.888 (88.8%), and a wavelength range of 2.5–20 µm at 35 °C (mW/cm^2^). The placebo garment (KYMIRA, Reading, UK) was 275 g/m^2^ and consisted of 75% Polyester and 25% elastane. The double-blind distribution resulted in two females and three males receiving the FIR garments and four females and one male receiving the placebo.

### 2.3. Anthropometrics and Initial Survey

The participants’ height, weight, and body composition were measured following standard protocols [17]. Their height was recorded to the nearest 1 mm using a portable stadiometer, and their weight was recorded to the nearest 0.1 kg with an electronic scale. Their body composition was assessed via skinfold thickness (bicep, tricep, subscapular, and suprailiac) and bioelectrical impedance measurements. Skinfolds were measured three times at each site with Lange skinfold calipers (Beta Technology, South Burlington, VT, USA), and the mean was used to calculate the body fat percentage and fat-free mass following Durnin and Womersley (1974) [18]. For the bioelectrical impedance measurements, participants refrained from alcohol for 24 h before the measurements, which were taken using an RJL Quantum X bioelectrical impedance unit (RJL Systems, Clinton Township, MI, USA). Standard BIA-103 equations (Brodie and Eston, 1992) [19] were applied to calculate their fat-free mass, body fat percentage, and fat mass.

### 2.4. Resistance Exercise Protocol

The participants completed a dynamic warm-up of their choosing, lasting between five and ten minutes. During the final part of the warm-up, the participants performed warm-up sets of back squats, gradually increasing the weight to 85% of their one-repetition maximum (1RM). This weight was selected due to its association with high muscular activation and its capacity to provide meaningful stress to the neuromuscular system, while not being so heavy as to severely limit the number of repetitions performed with the proper technique. The participants were then instructed to complete as many back squat repetitions as possible, stopping at two repetitions in reserve (RIRs) at 85% of their 1RM. This protocol has been shown in prior research to elicit acute neuromuscular fatigue and performance decrements, even when using a single high-intensity set (Pareja-Blanco, 2017) [20]. All the sets were performed under the supervision of a certified strength and conditioning coach acting as a spotter.

### 2.5. Subjective Questionnaires

At the completion of each squat protocol, the participants verbally rated their perceived exertion as a value between 1 and 10, using Borg’s CR-10 scale [21]. At the end of session 4, the participants were asked, “Which best describes your recovery”, and provided with three options to choose from: less than 24 h, between 24 and 48 h, and more than 48 h.

### 2.6. Countermovement Jump

Countermovement jumps (CMJs) were performed immediately before and after the squat protocol and at 24 and 48 h following the protocol. All the jumps were recorded using portable, uniaxial, dual force plates operating at 1000 Hz (ForceDecks, FDLite V.2, VALD, Brisbane, Australia). For each jump trial, the plates were zeroed and calibrated prior to the subject stepping onto them. The subjects then placed their hands on their hips and remained still while their body weight was recorded. The subjects were then instructed to keep their hands fixed on their hips and after a “3, 2, 1, JUMP” countdown were asked to jump as high as they could and land back on the force plates. The participants were instructed to reset their feet, resulting in a brief (~3 s) rest period between the jumps. Five jumps were recorded and used for analysis (one subject completed only four jumps at baseline). The metrics included for analysis were calculated from the raw force time series data using the proprietary software (ForceDecks Jump, V2.0.8997, VALD, Brisbane, Australia). All five jumps were used subsequently in the analyses described below.

The following countermovement jump (CMJ)-derived metrics, calculated using ForceDecks software (ForceDecks Jump, V2.0.8997, VALD, Brisbane, Australia), were included in the analysis to assess the participants’ neuromuscular status. The jump height (JH), calculated using the impulse momentum method, represented the vertical displacement achieved during the jump [22]. The modified reactive strength index (mRSI) was defined as the ratio of the jump height to the time spent in the eccentric phase, providing an insight into the participant’s explosive ability [23]. The takeoff velocity (TV) measured the velocity at the moment of takeoff and served as an indicator of participants’ readiness and freshness [24]. The force at zero velocity (F@0V) quantified the maximal force produced at the instant when the velocity was zero during the transition between the eccentric and concentric phases [25]. The eccentric rate of force development (ERFD) was the rate at which the force was developed during the eccentric phase of the CMJ [26]. The braking impulse (BI) was the total impulse generated during the deceleration phase, reflecting the ability to control and absorb the force [27]. The eccentric duration (ED) refers to the time spent in the eccentric phase of the movement [28]. These metrics collectively provided a detailed assessment of the participants’ neuromuscular performance and status.

### 2.7. Fatigue Biomarkers

Saliva samples were collected immediately before and after the squat protocol and at 24 and 48 h following the protocol. The participants were instructed to avoid eating, drinking (except water), and brushing their teeth within the 30 min prior to collection. Saliva was collected using a cotton swab device (Sarstedt Salivette^®^; Sarstedt Inc., Newton, NC, USA). The participants were directed to remove the sponge portion of the device and place it in their mouth until fully saturated (three minutes). The saturated sponge was subsequently placed back into the top portion of the device and then placed immediately into the freezer. The samples were kept at −20 °C until shipped with ice packs to Hyperion Biotechnologies Inc. (San Antonio, TX, USA) for analysis. The saliva samples were analyzed using validated immunoassay techniques in accordance with standard procedures [29]. The saliva samples were analyzed for the fatigue biomarker index (FBI) and myoglobin. The FBI is the log of the ratio of two salivary peptides associated with fatigue (the amino acid sequences GGHPPPP and ESPSLIA, with the former acting as the numerator in the FBI ratio). Previous research has reported that greater fatigue results in decreased GGHPPPP and increased ESPSLIA [29]. The FBI has been validated as being indicative of experienced physiological fatigue in a variety of contexts, including predicting military training outcomes [30], exercise [29], sleep deprivation [31], general fatigue [32], and chronic fatigue [33]. Lab testing performed by Hyperion was completed as per Michael et al. (2012) [29].

### 2.8. Statistical Analyses

A one way analysis of variance (ANOVA) was conducted to compare the sex differences in the reps completed and rate of perceived exertion, as well as to assess the differences in the reps completed and rate of perceived exertion between those wearing the FIR-emitting garment and those wearing the placebo. Mixed linear regression models (MLMs) were used to assess several aspects of the study due to the repeated measurements. First, each of the CMJ variables was used as the dependent variable in its own MLM, with pre-/post-squat (within session 2) as a binary independent variable and the subject as a random effect. If the post-squat value was lower than the pre-squat value, this would indicate that the subjects were fatigued by the squats. Then, to further examine the relationship between the force plate metrics and the effects of the time and tights, a linear mixed model was fit for each metric with that metric as the dependent variable, with the independent variables being the time (pre-squat prior to session 2 vs. 24 and 48 h post-squat), the tights (FIR vs. placebo), and the interaction between the time and tights. This process was carried out separately for data collected one and two days after the squat session. All the analyses were performed using the R statistical computing environment (R Core Team, 2024), and the mixed models were fit using the *lmer* package [34]. The effect sizes for the mixed linear models were calculated by standardizing each response variable and refitting the models. The binary predictors and their interaction were not standardized, resulting in “partially standardized coefficients” (Lorah, 2018) [35]. We used traditional levels of statistical significance throughout: 0.01 ≤ *p*-value < 0.05 indicated marginal significance, 0.001 ≤ *p* = value < 0.01 indicated significance, and *p*-value < 0.001 indicated high significance. These thresholds reflected widely used conventions for interpreting the statistical significance (Cohen, 1988) [36].

## 3. Results

**Figure 2:**
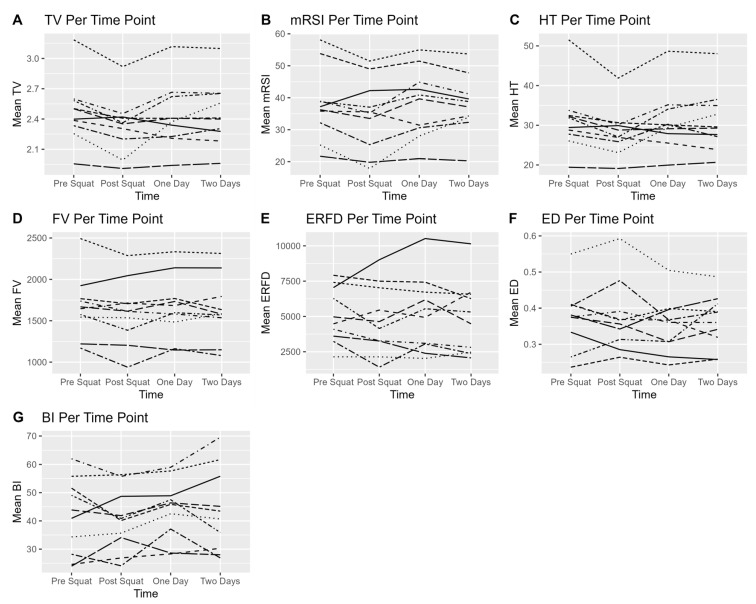
Changes in countermovement jump (CMJ) variables across four time points: baseline (pre-squat), immediately post-squat, 24 h post-squat, and 48 h post-squat. Each subplot (**A**–**G**) represents specific jump metric: (**A**) takeoff velocity, (**B**) modified reactive strength index (mRSI), (**C**) jump height, (**D**) force at zero velocity, (**E**) eccentric rate of force development (RFD), (**F**) eccentric duration, and (**G**) braking impulse.

Table 1 presents the demographic and descriptive statistics. Figure 2 demonstrates individual participants’ recovery over time based on their CMJ metrics. The results of the ANOVA show that the difference in the repetitions between females and males was near statistical significance (F(1,10) = 5.232, *p* = 0.05), though no significant difference in the RPE was observed (F(1,10) = 0.914, *p* = 0.367). The participants using the FIR garment reported a significantly higher RPE (F(1,10) = 12.800, *p* = 0.007).

### 3.1. Countermovement Jump

The pre- and post-squat countermovement jump performances in session 2 were significantly different for four variables. Specifically, the TV (*p* < 0.001), mRSI (*p* < 0.001), JH (*p* < 0.001), and F@0V (*p* = 0.005) were significantly lower on average post-squat. Though not statistically significant, the ERFD (*p* = 0.19) and BI (*p* = 0.59) were also lower post-squat. Conversely, the ED was slightly greater post-squat, though this was not statistically significant (*p* = 0.93).

Table 2 presents the mean values for each CMJ-derived metric at baseline (pre-squat) and one and two days post-squat protocol. The percent changes from the baseline are also included, enabling comparisons across metrics expressed in different units. For example, in the second row, the mean TV for participants wearing the placebo is shown. The percent change for the TV one day post-squat was calculated to be (2.42 − 2.50)/2.50 = −0.032, or −3.2%.

Table 3 and Table 4 summarize the results of the linear mixed-effects models. The interaction term in these models evaluated the relative effectiveness of the far-infrared (FIR) tights compared to the placebo tights (placebo condition). For instance, the estimated parameters in Table 3 indicate that for the modified reactive strength index (mRSI), the predicted mean baseline value for the placebo tights wearers was 40.40, which decreased to 39.53 post-squat. In contrast, the FIR tights wearers had a predicted baseline value of 35.19 and a post-squat value of 37.57. The interaction term suggests a statistically significant improvement in the mRSI with the FIR tights post-squat. Table 4 highlights significant interaction effects for the mRSI, JH, F@0V, and ERFD when comparing the values at baseline to those two days post-squat. These findings underscore the differential impact of the FIR tights on the neuromuscular performance recovery metrics relative to that of the placebo.

### 3.2. Fatigue Biomarkers

The first model included an effect for the garment (FIR vs. placebo) pre-squat to determine if there were any a priori differences between the groups. The second model included effects for the garment, the time (pre-squat vs. one day after the squat protocol), and the interaction between the garment and time. The third model was similar to the second, but with the time variable encompassing the values at baseline vs. two days after the squat session. All the effects in all three models were not statistically significant; thus, there was no difference in the fatigue index across the time points or garments.

## 4. Discussion

This study examined the efficacy of far-infrared garments for improving the objective and subjective recovery of an active cohort following a resistance exercise bout. The results suggest that FIR garments enhanced neuromuscular recovery, as demonstrated by the CMJ performance, and the perceived recovery duration relative to the placebo. However, the recovery status based on fatigue biomarkers was not influenced by the use of FIR garments. As such, our results suggest that FIR garments may pose benefits to recovery following short bouts of resistance exercise among the recreationally active, though further research is required to determine their efficacy in recovery from other forms of activity, such as maximal aerobic and anaerobic training.

The primary finding of this study suggests that neuromuscular recovery was enhanced following a resistance exercise bout with the use of a far-infrared (FIR) garment. While limited changes were observed after 24 h of recovery, with only the mRSI (*p* < 0.05) showing significant improvement, several CMJ metrics demonstrated significant improvements after 48 h, including the force at zero velocity (*p* < 0.001), eccentric RFD (*p* < 0.001), takeoff velocity (*p* < 0.05), jump height (*p* < 0.001), and mRSI (*p* < 0.001). The delayed improvements likely reflect the cumulative benefits of FIR garments on recovery processes, such as enhanced peripheral blood flow, improved tissue oxygenation, and the accelerated removal of metabolic byproducts [37], which can aid muscle contractility and force production. These effects may align with the natural recovery timeline, where the resolution of exercise-induced muscle damage and inflammation typically peaks between 24 and 48 h post-exercise [38]. The earlier improvement in the mRSI suggests that explosive strength and the stretch-shortening cycle efficiency may be more sensitive to an FIR intervention in the initial stages of recovery [39]. The observed enhancements in neuromuscular function highlight the potential of FIR garments to accelerate recovery by optimizing tissue perfusion, metabolic clearance, and neuromuscular readiness, with the most pronounced effects emerging 48 h post-exercise.

In addition to improved objective measures of recovery, the participants also reported a shorter perceived recovery duration when using the FIR garments. In line with the objective measures, the participants who wore the FIR garments reported feeling that they had recovered within 24–48 h, whereas those who wore the placebo typically reported recovery taking longer than 48 h. Additionally, 54% of the participants reported feeling that they had recovered more when using the FIR garment, while 18% preferred the placebo. The remaining participants found the FIR garment and placebo to provide similar perceived recovery. These subjective perceptions align closely with the time course of recovery observed in the CMJ metrics, where significant improvements occurred primarily at the 48 h mark. The perceived recovery is particularly important for recreationally active individuals, as it can influence their readiness to resume physical activity, reduce feelings of fatigue, and promote adherence to regular exercise routines [40]. The improved perceptions of recovery when using FIR garments may be linked to physiological mechanisms, such as enhanced blood flow, reduced muscle soreness, and the accelerated clearance of metabolic byproducts [37]. Additionally, psychological factors, such as a belief in the effectiveness of the garment and a sense of improved comfort [41], may have contributed to these findings. The alignment between the objective improvements in the CMJ metrics and the subjective perceptions of recovery suggests that FIR garments may offer meaningful benefits for enhancing recovery and supporting consistent physical activity among recreationally active individuals.

The findings of this study align with and extend existing research on the use of far-infrared (FIR) garments for recovery following resistance exercise, particularly in populations that engage in recreational physical activity. Previous studies have demonstrated that FIR garments can enhance recovery by improving the peripheral blood flow, reducing muscle soreness, and accelerating the clearance of metabolic byproducts [37]. For instance, Loturco et al. (2016) [8] reported reduced delayed-onset muscle soreness (DOMS) and improved muscle function following strenuous exercise with FIR garment use, outcomes that are particularly relevant for recreationally active individuals looking to return to activity sooner and with less discomfort. Similarly, Leung et al. (2013) [37] highlighted improved microcirculation as a potential mechanism facilitating muscle recovery and contractility. In the present study, significant improvements in neuromuscular measures were observed primarily at 48 h post-exercise, suggesting that FIR garments may provide cumulative benefits over time. This aligns with prior research indicating that FIR garments’ effects are most pronounced 24–48 h post-exercise [8]. Importantly, for recreationally active individuals, enhanced recovery and reduced fatigue may encourage more consistent participation in physical activity and exercise programs. Overall, these findings support the use of FIR garments as a practical and effective tool for improving both objective recovery metrics and subjective perceptions of recovery following resistance exercise in non-elite, active populations.

Though improvements in the CMJ metrics were observed, this study was unable to detect changes in saliva-based fatigue biomarkers, such as the FBI and myoglobin. Previous research validating the use of saliva-based fatigue biomarkers involved participants completing 10 h of activity [29]—a far greater magnitude of stress than that in the present study. While the resistance exercise protocol was sufficient to induce observable neuromuscular fatigue, as evidenced by reductions in the CMJ performance immediately post-exercise, the absence of significant changes in the salivary fatigue biomarkers suggests that the physiological load may not have been sufficient to provoke a systemic biochemical response (Papacosta & Nasis, 2011) [42]. This aligns with prior research showing that the FBI and myoglobin are more responsive to prolonged or high-volume physical stress (Brancaccio, Maffulli & Limongelli, 2007; Hecksteden et al., 2017) [43,44]. It is likely that the stress experienced by the participants was below the physiological threshold needed to elicit a detectable biomarker response. This highlights a key limitation of this study: the short, largely anaerobic nature of the exercise protocol, which, while sufficient to induce neuromuscular changes as reflected in the CMJ metrics, was not stressful enough to trigger measurable biomarker alterations. It is also possible that the selected biomarkers, while validated in military (Nindl et al., 2002) [45] and endurance contexts (Banfi et al., 2006; Halson & Jeukendrup, 2004) [46,47], may be less sensitive to isolated bouts of resistance training. Future studies should consider complementing the use of such biomarkers with muscle-damage-specific markers (e.g., creatine kinase) or use longer-duration resistance or metabolic protocols that induce more systemic fatigue. Additionally, the small sample size limits the generalizability of the findings. A larger sample size, combined with a crossover design, would provide greater statistical power and better elucidate the impact of FIR garments on recovery. Future research should consider employing longer or more physically demanding exercise protocols to induce sufficient fatigue for biomarker detection or focus on alternative fatigue measures, such as the neuromuscular performance (e.g., CMJ metrics), or more sensitive biomarker tests better suited to shorter exercise challenges.

## 5. Conclusions

In conclusion, this study demonstrates that FIR garments can enhance recovery following a resistance exercise bout in a recreationally active population, as evidenced by improvements in their neuromuscular performance metrics and subjective perceptions of recovery. Significant improvements in the CMJ metrics were primarily observed 48 h post-exercise, suggesting that FIR garments provide cumulative benefits for recovery. The alignment of these objective findings with participants’ reports of feeling that they had recovered more within 24–48 h supports the potential of FIR garments to facilitate both objective and subjective recovery. However, the lack of changes in the saliva-based fatigue biomarkers highlights the limitations of using short, anaerobic exercise challenges to elicit biomarker responses. Future research should consider using longer, more demanding exercise protocols or alternative fatigue markers to better understand the full scope of FIR garments’ impact on recovery. Nonetheless, the results suggest that FIR garments may be a valuable tool for improving recovery and supporting consistent physical activity participation in recreationally active individuals.

## Figures and Tables

**Table 1:** Descriptive statistics (mean ± standard deviation).

	Overall (N = 10)	FIR (N = 5)	Placebo (N = 5)
Age (yrs)	20.7 ± 3.2	21.8 ± 4.2	19.6 ± 1.5
Height (cm)	171.4 ± 8.8	174.7 ± 6.6	168.0 ± 10.0
Weight (kg)	75.6 ± 13.5	79.9 ± 8.8	71.4 ± 17.0
Fat % (Skinfolds)	23.2 ± 6.8	19.9 ± 6.5	26.5 ± 5.8
Fat % (BIA)	28.5 ± 5.1	24.9 ± 3.9	32.0 ± 3.6
SMM (BIA, kg)	25.7 ± 8.5	31.8 ± 5.0	19.7 ± 6.7
Lifting Experience (yrs)	4.4 ± 2.4	3.8 ± 2.1	4.9 ± 2.9
1 Rep Max (kg)	274 ± 91	283 ± 67	266 ± 117
~85% of 1RM (kg)	234 ± 77	241 ± 55	226 ± 100.0
Reps Completed	14.7 ± 2.2	12.4 ± 3.2	13.2 ± 3.0
RPE	8.8 ± 0.4	8.3 ± 0.3	8.7 ± 0.5

**Table 2:** Linear mixed model parameter estimates for pre-squat values vs. values one day after squat session. Differences and percent changes in CMJ metrics broken down by time point and FIR/placebo condition.

Metric	Garment	Pre-Squat	1 Day After	Percent Change from Pre-Squat Value	2 Days After	Percent Change from Pre-Squat Value
Takeoff Velocity	FIR	2.44	2.44	0.00	2.47	1.23
	Placebo	2.50	2.42	−3.20	2.43	−2.80
mRSI	FIR	35.19	37.56	6.73	37.57	6.76
	Placebo	40.75	39.53	−2.99	38.23	−6.18
Jump Height	FIR	29.99	30.41	1.40	31.16	3.90
	Placebo	32.88	31.62	−3.83	30.94	−5.90
Force @ Zero Velocity	FIR	1685.04	1697.26	0.72	1724.36	2.33
	Placebo	1684.25	1628.77	−3.29	1550.77	−7.93
Eccentric RFD	FIR	4806.40	5223.14	8.67	5491.60	14.26
	Placebo	5520.21	5155.06	−6.61	4350.41	−21.19
Eccentric Duration	FIR	0.39	0.36	−7.69	0.37	−5.13
	Placebo	0.36	0.34	−5.56	0.36	0.00
Braking Impulse	FIR	42.17	45.25	7.30	46.44	10.13
	Placebo	41.21	43.13	4.66	41.04	−0.41

**Table 3:** Linear mixed model parameter estimates and effect sizes (ESs) for pre-squat values vs. values one day after squat session.

Metric	Intercept (ES)	BETA_Time (Post) (ES)	BETA_Tights (FIR) (ES)	BETA_Interaction (ES)
Takeoff Velocity	2.50 *** (0.14)	−0.08 * (−0.24)	−0.05 (−0.16)	0.07 (0.23)
mRSI	40.40 *** (0.20)	−0.87 (−0.08)	−5.21 (−0.47)	3.25 * (0.30)
Jump Height	32.76 *** (0.20)	−1.14 * (−0.15)	−2.77 (−0.36)	1.56 (0.20)
Force @ Zero Velocity	1664.44 *** (−0.03)	−35.68 (−0.10)	20.59 (0.06)	47.90 (0.13)
Eccentric RFD	5433.32 *** (0.11)	−278.26 (−0.12)	−626.92 (−0.26)	695.00 (0.29)
Eccentric Duration	0.36 *** (−0.02)	−0.02 (−0.21)	0.03 (0.29)	−0.01 (−0.08)
Braking Impulse	40.86 *** (−0.14)	2.27 (0.15)	1.31 (0.09)	0.81 (0.05)

Note: “*” indicates 0.01 ≤ *p*-value < 0.05; and “***” indicates *p*-value < 0.001.

**Table 4:** Linear mixed model parameter estimates and effect sizes (ESs) for pre-squat values vs. values two days after squat session.

Metric	Intercept (ES)	BETA_Time (Post) (ES)	BETA_Tights (FIR) (ES)	BETA_Interaction (ES)
Takeoff Velocity	2.50 *** (0.12)	−0.06 * (−0.20)	−0.05 (−0.17)	0.08 * (0.27)
mRSI	40.47 *** (0.26)	−2.24 * (−0.22)	−5.28 (−0.53)	4.61 *** (0.46)
Jump Height	32.70 *** (0.19)	−1.76 ** (−0.23)	−2.71 (−0.35)	2.93 ** (0.38)
Force @ Zero Velocity	1664.34 *** (0.01)	−113.57 *** (−0.31)	20.69 (0.06)	152.89 *** (0.42)
Eccentric RFD	5436.55 *** (0.17)	−1086.15 ** (−0.45)	−630.15 (−0.26)	1771.34 *** (−0.22)
Eccentric Duration	0.36 *** (−0.09)	0.00 (0.00)	0.03 (0.29)	−0.02 (−0.22)
Braking Impulse	40.68 *** (−0.13)	0.37 (0.02)	1.49 (0.10)	3.91 (0.25)

Note: “*” indicates 0.01 ≤ *p*-value < 0.05; “**” indicates 0.001 ≤ *p*-value < 0.01; and “***” indicates *p*-value < 0.001.

## Data Availability

The original contributions presented in this study are included in the article. Further inquiries can be directed to the corresponding author(s).
